# Design, fabrication and testing of 3D printed smartphone-based device for collection of intrinsic fluorescence from human cervix

**DOI:** 10.1038/s41598-022-15007-x

**Published:** 2022-07-01

**Authors:** Shivam Shukla, Amar Nath Sah, Diganta Hatiboruah, Shikha Ahirwar, Pabitra Nath, Asima Pradhan

**Affiliations:** 1 Center for Lasers and Photonics, IIT Kanpur, Kanpur, 208016 India; 2 Department of Physics, IIT Kanpur, Kanpur, 208016 India; 3 Department of Biological sciences and Bioengineering, IIT Kanpur, Kanpur, 208016 India; 4 Department of Physics, Tezpur University, Tezpur, 784028 India; 5 PhotoSpIMeDx Pvt. Ltd., SIIC, IIT Kanpur, Kanpur, 208016 India

**Keywords:** Gynaecological cancer, Optical techniques, Preclinical research, Optical sensors

## Abstract

Fluorescence spectroscopy has the potential to identify discriminatory signatures, crucial for early diagnosis of cervical cancer. We demonstrate here the design, fabrication and testing of a 3D printed smartphone based spectroscopic device. Polarized fluorescence and elastic scattering spectra are captured through the device using a 405 nm laser and a white LED source respectively. The device has been calibrated by comparison of spectra of standard fluorophores (Flavin adenine dinucleotide, fluorescein, rhodamine, and porphyrin) with the corresponding spectra collected from a commercial spectrometer. A few cervical tissue spectra have also been captured for proof of its applicability as a portable, standalone device for the collection of intrinsic fluorescence spectra from human cervix.

## Introduction

In recent years, a rapid increase has been observed in cervical cancer cases with an estimated 1.04 lakh new cases and 60,000 deaths reported by International Agency for Research on Cancer (IARC) in India in 2020^1^. Cervical cancer can be categorized, with increasing abnormality, into three grades: CINI, CINII and CINIII (Cervical intraepithelial neoplasia)^2^. The disease can be cured if diagnosed at an early stage. There are several conventional methods of diagnosis like Pap smear test, liquid based cytology, colposcopy, and tissue biopsy with histopathology which is considered as gold standard. Although, aforementioned techniques have been widely used for decades, they generally fail to adequately serve the purpose due to either low sensitivity or specificity and lack of real time analysis. Optical spectroscopy, especially fluorescence spectroscopy, has the potential to monitor subtle morphological and biochemical changes occurring in the tissue with disease progression, based on the response of different fluorophores (NADH, FAD, collagen, and porphyrin) present in the layered cervical tissue structure. Over the last several decades, many researchers have applied fluorescence spectroscopy to examine abnormality of different grades in different type of tissues and in many other similar applications^3–7^. Britton Chance and his group reported the very first use of this technique to collect autofluorescence of Flavin/NADH from cells^8^. Alfano initiated this in biological tissues by observing autofluorescence from rat tissue^9^. Since then, many group worked on different aspects of fluorescence spectroscopy such as polarization, synchronous, spatially resolved fluorescence spectroscopy. Alfano and Pradhan et al. used fluorescence spectroscopy for the diagnosis of normal and cancerous breast tissues^10^. Ramanujam et al. collected fluorescence spectra from various cervical sites in an in-vivo study using a portable fluorimeter and analyzed it further with multivariate statistical algorithm^11^. Chang et al. was able to discriminate high grade cervical precancers from normal tissues by combining reflectance and fluorescence spectroscopy^12^. The research then geared towards clinical devices. Liu et al. reported the development of a portable point detection system based on intrinsic fluorescence redox ratio for brain cancer diagnosis^13^. The efficacy of fluorescence spectroscopy was parallelly examined by researchers. Majumdar et al. and Fransisco et al. tested oral cancer patients to discriminate different grades^14,15^. Jermyn et al. employed intrinsic fluorescence spectroscopy in a multimodal approach for the detection of brain, lung, colon, and skin cancers with good accuracy^16^. Meena and group discriminated different grades of cancer using an in-house developed intrinsic fluorescence-based probe^17^. Kumar et al. compared the efficiency of fluorescence and Stokes shift spectroscopy for the detection of oral cancer using human saliva as a diagnostic medium^18^.

However, most of the previously mentioned fluorescence spectroscopy-based systems are limited to controlled laboratory environment due to their bulky size and requirements of expensive light sources and detectors. WHO has recommended to build systems which are affordable, sensitive, specific, user-friendly, rapid and robust, equipment free and deliverable to end users. In recent years, with increase in the number of users (approx. 8 billion), smartphone has become a primary tool to build such portable, light weight and low-cost spectroscopic devices^19–23^. It works on wireless platforms and is equipped with advanced technology for computation, display and sensing. Breslauer et al. built a mobile phone light microscope to capture the images of *P. falciparum* infected red blood cells and *M. tuberculosis* infected sputum samples^24^. The integrated lens and image sensor of the smartphone were transformed into a microscope and spectrometer by Smith and the group to image both stained, unstained blood smears and acquire the fluorescence spectrum of Rhodamine 6G^25^. Gallegos et al. reported a very first use of smartphone as a spectrometer to detect shifts in the resonant wavelength of a photonic crystal label-free biosensor^26^. Yu et al. incorporated a smartphone fluorimeter for the detection of a specific nucleic acid sequence and compared its performance with conventional fluorimeter^27^. An optical fiber-based smartphone spectrometer was developed by Hossain et al. for the food quality monitoring by characterizing changes in pigments of an apple via absorption spectroscopy^28^. Das et al. also worked with an ultra-portable, standalone smartphone spectrometer for the rapid and non-destructive testing of fruit ripeness by capturing UV fluorescence of chlorophyll on smartphone^29^. A low-cost, robust, and portable smartphone fluoride sensor was showcased by Hussain et al. to detect and analyze the fluoride concentration level in drinking water. For this purpose, they utilized LED flash of smartphone as light source and ALS (Ambient light sensor) of smartphone as light intensity detector^30,31^. Ding et al. demonstrated a smartphone-based spectrometer using specially designed wavelength calibration and intensity correction methods to acquire better accuracy for absorption and fluorescence spectrum of fluorescent dyes^32^. Hong et al. recently described a dual-modality fiber-optic micro-endoscope that integrates diffuse reflectance spectroscopy and fluorescence imaging into a smartphone platform for quantifying physiological and morphological properties of epithelial tissues. Preliminary in-vivo measurements done on different parts of healthy human oral tissues were also shown to proof its capability^33^.

In this study, we have worked on the development of a portable, easy to handle smartphone-based device which works on the extraction of intrinsic fluorescence from tissue samples using the measured polarized fluorescence and elastic scattering spectra on the smartphone camera. Intrinsic fluorescence is different from the bulk fluorescence collected from the tissue as it is free from wavelength dependent scattering and absorption properties of a highly turbid medium like tissue. Biswal et al. presented an experimental approach for the removal of distortions related to scattering and absorption from the bulk fluorescence spectra. They were able to recover the actual intensity and line shape of fluorescence from the fluorophores present in a variety of tissue mimicking phantoms having different scattering and absorption properties^34^. Devi et al. incorporated the same approach to calculate intrinsic fluorescence for the classification of different grades of cervical cancer using a standard fluorimeter system^35^. Subsequently, a device was designed, fabricated and tested using a miniature spectrometer and laser systems by Meena et al. for the collection of intrinsic fluorescence spectra^17^. The encouraging results from this probe prompted us to improve the system to a more compact and easy to handle device using smartphone based technology.

## Materials and methods

### Design and details of the smartphone-based device

**Figure 1:**
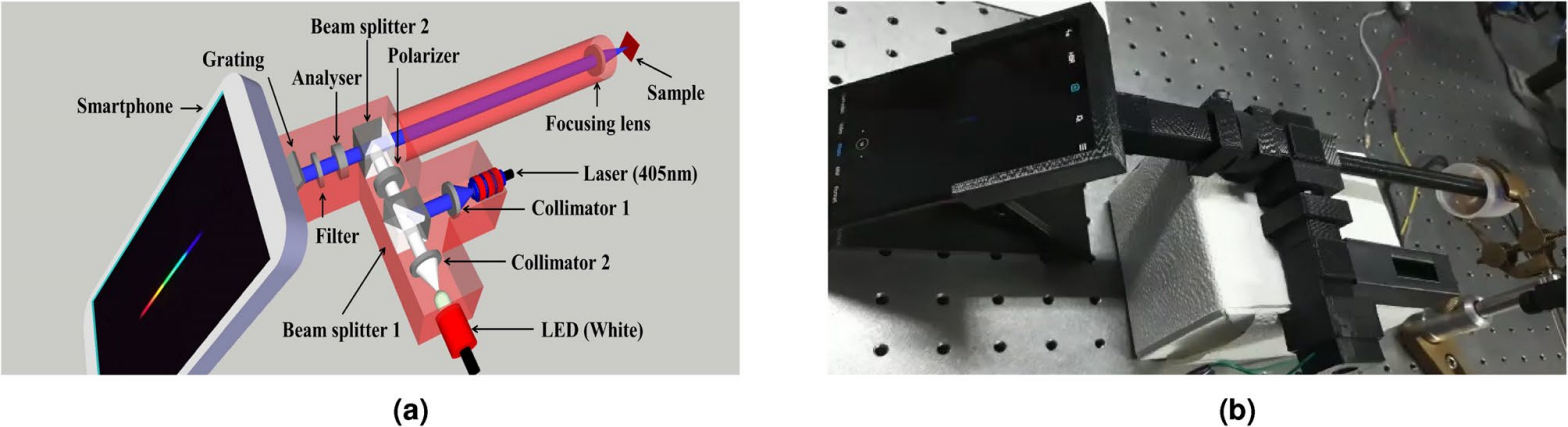
(**a**) Schematic (drawn using an open access software, SketchUp 2014, www.sketchup.com ) and (**b**) working set-up of smartphone-based polarized fluorescence detection system.

The proposed device is an upgraded version of a previously in-house developed intrinsic fluorescence detection probe using spectrometer as a detector^17,36,37^. The device can be divided into two chambers: input chamber and output chamber as shown by the schematic and working set-up of the device in Fig. 1a,b.

**Input chamber:** It consists of two light sources: a 405nm laser source (Adlabs Instruments, New Delhi) for fluorescence collection and a miniature 10 W white LED (CREE XM-L T6) for elastic scattering spectra, one pair of 12 mm focal length collimating lenses (LA1576, Thorlabs) for each source, a 25.4 mm diameter Glan-Thompson polarizer (10GT04AR.14, Newport) to pass polarized light and a 10 mm non-polarizing cubic beam splitter (BS010, Thorlabs) for the simultaneous reflection and transmission of the incident light.

**Output chamber:** It has several components such as a beam splitter (BS010) to allow the input light to the sample and pass the back-projected fluorescence signal to analyzer (10GT04AR.14), a 13 cm long narrow cylindrical tube for easy access to the surface of the cervix through the vagina, a 450 nm 12.5 mm diameter long pass filter (OD 4.0 LPF, Edmund optics) to eliminate the incident light and at the end, a detector assembly which comprises of a 12.7 mm $\times$ 12.7 mm visible transmission grating (GT13-12, Thorlabs), with 1200 grooves/mm, to convert the collected polarized fluorescence and elastic scattering intensity into a visible spectrum and a Redmi K20 smartphone to record and analyze the resultant spectrum.

Grating has been arranged at a 45$^{\circ }$ angle with respect to the incoming light and a smartphone is placed parallel to the grating so that we only get the 1st order spectrum of the grating at the CMOS camera location. The overall dimension (L $\times$ B $\times$ H) of the device is approximately 26 cm $\times$ 15 cm $\times$ 4 cm and average weight is 300 g (here we have excluded the height $\sim 15$ cm and weight $\sim 90$ g of the smartphone). Ports for the light sources and all the holders for other small sized optical components have been designed on ZW3D, a 3D CAD designing software, and printed via a 3D printing machine (Raise 3D). A biodegradable and non-fluorescent PLA material is used for printing all these components, keeping in mind the compatibility of the device for biomedical applications.

### Image processing and device calibration

**Figure 2:**
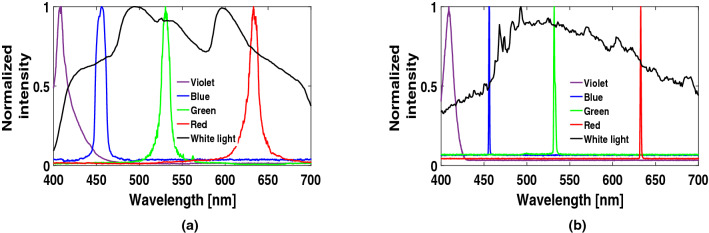
Spectra of four different lasers and Xe-lamp from (**a**) smartphone and (**b**) spectrometer.

In a spectroscopic study, one works on the entire spectral range (400–700 nm in our case) to visualize the resultant spectrum in a more standard way. So, it is crucial to capture the resultant spectral image from a grating in a RAW image format which produces a more reliable unprocessed scientific data than a typical JPEG image format. In literature, one finds very few researchers who have discussed about employing RAW image format to process the mobile camera images^38,39^. For our purpose, we have compared the performance and accuracy of both the formats and chosen the RAW image format. Calibration of the device has been done using four different laser sources, Violet (405 nm), Blue (456 nm), Green (532 nm) and Red (633 nm), to cover the entire visible range (400–700 nm). Spectra of all these lasers and that of a continuous Xenon lamp light source, collected both from the smartphone and a commercial spectrometer (USB 4000, Ocean Optics), are shown for comparison in Fig. 2a,b^37^.

**Figure 3:**
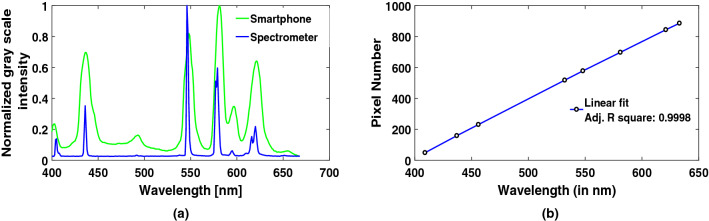
(**a**) Spectra of mercury lamp from smartphone and spectrometer, (**b**) Pixel v/s wavelength curve.

**Figure 4:**
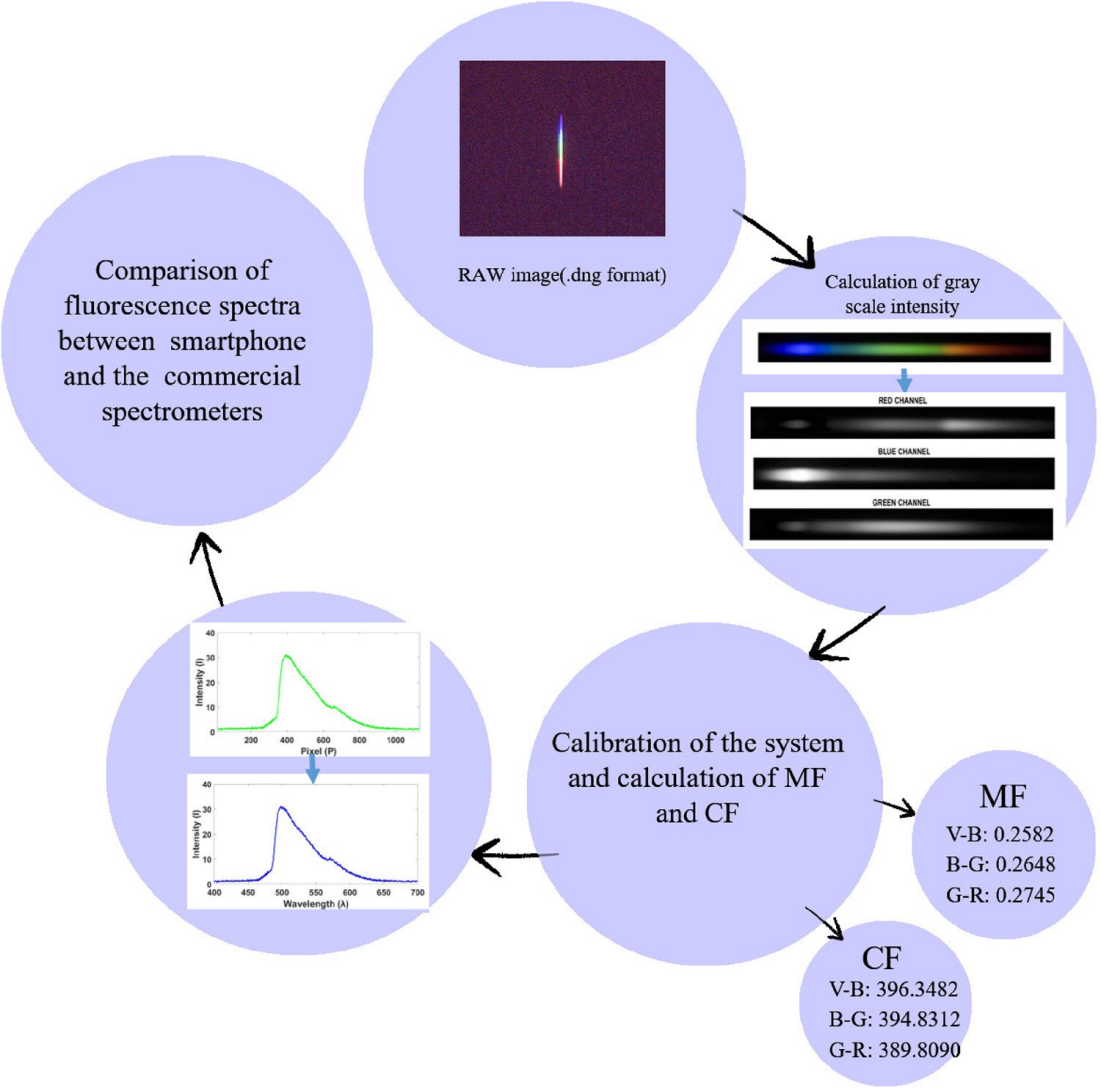
Flow chart explaining pixel to wavelength conversion and calibration process.

A two-step conversion method, explained in supplementary material, is employed here to convert observed pixel values into the corresponding wavelength values using the resultant values of MF (Multiplication factor) and CF (Constant factor). A comparison of mercury lamp spectra for known multiple peaks between our device and the commercial spectrometer have also been shown along with pixel to wavelength relationship curve to cross verify the calibration process (Fig. 3a,b). A flow chart related to image processing and wavelength calibration procedure has been showcased in Fig. 4.

### Intrinsic fluorescence

Intrinsic fluorescence (IF), as mentioned in introduction section, is extracted through a mathematical equation, modeled by Biswal et al.^34^, given as:

1 $$\begin{aligned} IF = \frac{[Ivv(\lambda )-G(\lambda )*Ivh(\lambda )]fl}{[Ivv(\lambda )-G(\lambda )*Ivh(\lambda )]scat}, \end{aligned}$$

where, Ivv($\lambda$) and Ivh($\lambda$) are intensities of co and cross polarized signals, G($\lambda$) is the ratio of the sensitivity of the proposed system for vertically and horizontally polarized light. Here, the subscripts ‘fl’ and ‘scat’ notify the fluorescence and elastic scattering respectively.

The polarized fluorescence and elastic scattering spectra, which are free from diffuse elastic scattering effect, are obtained by eliminating the cross-polarized parts of both the spectra from their respective co-polarized parts. To reduce the absorption effects in the output polarized spectra, a ratio is taken between the resultant fluorescence and elastic scattering spectra. This intrinsic fluorescence extraction scheme has been experimentally validated by several groups on tissue mimicking phantoms and in-vitro cervical tissue samples^17,35,40–42^.

### Samples for testing and validation

Some fluorescent dyes such as FAD, Fluorescein, Rhodamine 6G and Protoporphyrin, of known concentrations, have been used in this study for testing and validation of the proposed device and also to examine its efficiency in comparison with a commercial spectrometer. A few cervical tissue samples, provided by GSVM medical college, Kanpur, have also been tested with this device to evaluate its performance for such complex media. These cervix samples, freshly resected in surgery or biopsy, are first stored in ice for transportation from the hospital to our lab and within 5 h of surgery all the experiments are performed in the lab. Samples are then preserved in a formaldehyde solution and sent back to the hospital for histopathology. This whole study was reviewed and approved by the ethics committee of medical college. All experiments were carried out in accordance with relevant guidelines and regulations, and informed consent was obtained from all patients and/or their local guardian(s) by the doctors.

### Patent

A patent entitled “Smartphone based polarized fluorescence spectroscopic device for early detection of cervical cancer” with application number 202111006127 has been successfully filed.

## Results

The proposed device is capable of capturing the changes occurring in the spectra of endogenous fluorophores present in the cervical tissue. We have recorded spectra of some known fluorophores to validate the resultant polarized fluorescence spectra from the present system. A few cervical tissue samples have also been tested in-vitro and eventually, the generated fluorescence spectra have been compared with spectra from a commercial spectrometer. To record raw images (.dng format) we have employed a free camera app called Open Camera, available on Google play store, with ISO value and exposure time parameters fixed at 100 and 1 s respectively. These images are then processed through Adobe Photoshop software and MATLAB to convert them into a readable .tiff format and get the RGB values corresponding to each pixel value. Gray scale intensity (GSI) is finally plotted against wavelength to represent the resultant spectrum.

### Phantom study

**Figure 5:**
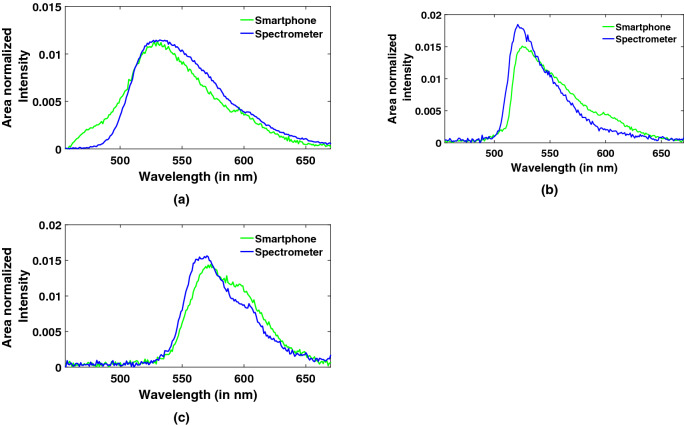
Comparison between smartphone and spectrometer for fluorescence spectra of (**a**) FAD, (**b**) Fluorescein, and (**c**) Rhodamine.

Liquid phantoms of some known fluorophores like FAD, rhodamine and fluorescein (C $\sim 125$ micro-molar) have been prepared to test the efficacy of the device for collecting the fluorescence spectra. All the solutions are prepared in our laboratory for testing. The resultant spectra are then compared with the spectra from USB4000 spectrometer as shown in Fig. 5a–c. From these plots, one can clearly deduce that there is quite a good match between all the smartphone spectra and the corresponding spectra from spectrometer with a slight broadening and a minimal shift of approx. 1–2 nm. All these plotted spectra are just the raw response of the smartphone camera without any further processing of the recorded images.

**Figure 6:**
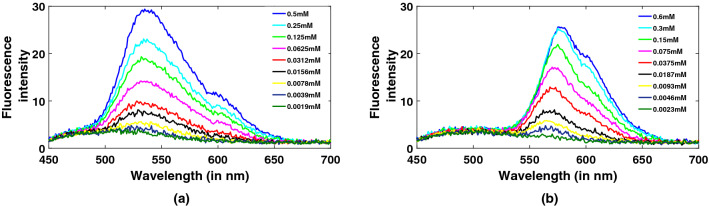
Fluorescence spectra of (**a**) FAD and (**b**) Rhodamine for different concentrations from smartphone-based prototype.

To evaluate the limit of detection (LoD) of the present system, spectra of FAD and rhodamine 6G (R6G) of different concentrations, 1.9-500 micro-molar (FAD) and 2.3-600 micro-molar (R6G), are recorded. Peak value is 532 nm for FAD and 572 nm for R6G. Peak values decrease with decrease in concentrations in both the fluorophore solutions as can be clearly seen in Fig. 6a,b. Their respective LoDs are thus estimated to be 3.9 and 4.6 micro-molar.

### In-vitro study on cervical tissue samples

**Figure 7:**
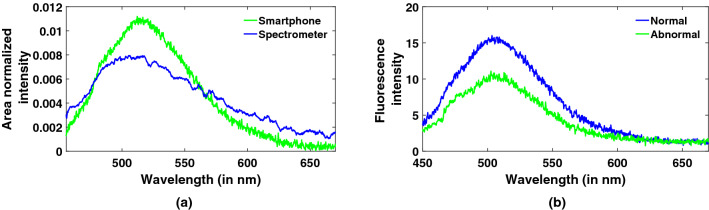
Typical plots of (**a**) polarized fluorescence spectra from smartphone and spectrometer, (**b**) normal and abnormal tissue spectra collected from smartphone based spectrometer.

**Figure 8:**
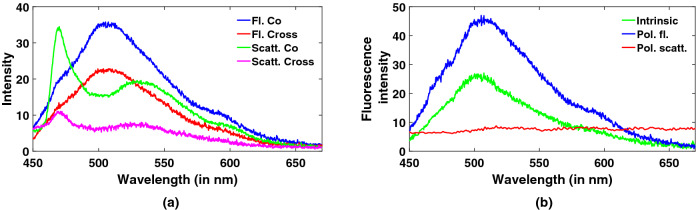
Typical plots of (**a**) co and cross polarized fluorescence and elastic scattering spectra and (**b**) polarized fluorescence, polarized scattering and intrinsic fluorescence spectra.

A few cervical tissue samples, provided by GSVM medical college, have been tested with our system. Images are collected from four sites of each sample following a clockwise pattern such as 12 o’clock, 3, 6 and 9 o’clock to observe changes within a particular sample^17^. Co and cross polarized fluorescence and polarized elastic scattering spectra are recorded by rotating the position of the analyzer. Figure 7a,b depict comparisons between polarized fluorescence spectra of smartphone and spectrometer, and spectra of normal and abnormal cervical tissue samples collected from smartphone. One can notice good discrimination between normal and abnormal fluorescence spectra in terms of decrease in intensity as well as a slight shift towards blue region. This can be explained as changes due to decrease in FAD fluorescence and increase in NADH peak respectively with increasing abnormality in cervical tissue samples. Intrinsic fluorescence is then extracted using Eq. (1) mentioned in materials and methods section. Comparisons of co and cross polarized fluorescence and elastic scattering spectra, and polarized fluorescence, polarized scattering and intrinsic fluorescence spectra for a cervical tissue sample have been showcased in Fig. 8a,b. A peak around 510 nm renders the presence of FAD in cervical tissue and a characteristic dip is also observed near 580 nm which displays the non-linear response of CMOS camera to wavelength. It is obvious from these plots that the resultant intrinsic fluorescence indeed eliminates the effect of spectral broadening and absorption dips present in the polarized fluorescence spectra.

## Discussion

In this study, we have demonstrated the design and testing of a portable, low-cost, user-friendly device which combines several small optical components and smartphone on a single platform to extract polarized fluorescence and polarized elastic scattering spectral images from samples. As we have mentioned earlier, this device is a miniaturized version of a previously fabricated and tested fluorescence detection probe. The present system replaces all the bulky electronics like laptop and spectrometer with mobile phone. Also, the external light sources like 405 nm laser with its driving unit and Xenon lamp source have been replaced by a small laser diode and white LED respectively. Designing a compact system with multiple small scale optical components is a tough task. To develop our system for fluorescence measurements, we initially worked on an open set-up and then optimized it for a closed environment. Other major challenge is the unavailability of research articles specially focused on involvement of smartphone-based spectrometer on biomedical applications. Hussain et al. in a review paper discussed about the recent development in smartphone based multimodal spectroscopic systems for biomedical applications and also the potential solutions for future applications in this field^43^. In spectroscopic measurements, for classification purpose or identification of some biomarkers, one needs to analyze the whole spectrum, i.e., 400–700 nm. Due to non-linear response of CMOS sensor for lights of different intensities and wavelengths, a set of correction factors is required, but still there will be a possibility of some spectral broadening and shift in the resultant spectra. Therefore, we have opted a RAW image format to eliminate the extensive use of multiple correction factors and get satisfactory spectra for our study. The proposed system has been calibrated using several known fluorescent dyes and the resultant spectra are in good agreement with the spectra of commercially available systems. Since this device records the native polarized fluorescence of samples in back-scattering mode, it is highly suitable for applications related to various biological tissues like cervical, oral, and skin. Pixel to wavelength conversion has been done using four laser sources (Violet, Blue, Green and Red) in visible range covering the entire region from 400–700 nm. This wavelength calibration is a two-step process, (1) To calculate multiplication factor (MF), and then (2) to add a constant factor (CF), explained in supplementary material using Supplementary Eq. S2 and Supplementary Eq. S3, for each wavelength window. Many researchers have just employed two LED/ laser pointers/lasers for the calibration process but for such kind of spectral analysis one should try to cover the whole visible region so that one gets one-to-one correspondence between pixel and wavelength values. As a prime application of the present device for in-vivo studies, it has been tested in-vitro on a few cervical tissue samples to check its performance in comparison to the standard spectrometers. The resultant spectra render promising results for both the polarized fluorescence and intrinsic fluorescence case. The performance of the device can be further improved by increasing the data size. Further, machine learning based algorithms would help in the classification of different categories of the samples. Also, from the spectra, one can clearly deduce that the response of the smartphone for various fluorescent dyes and tissue samples have been affected due to non-linear response of the CMOS sensor as seen in the form of slight broadening and characteristic dips. Intrinsic fluorescence has however, resolved these spectra related issues upto a great extent but this can be further improved by optimizing the conversion mechanism of spectra generated from the grating to a typical intensity versus wavelength curve in a more generalized way.

## Conclusion

In summary, a light weight, low cost, and easy to handle device based on a wireless platform, equipped with advanced technology, has been designed, fabricated, and successfully tested on a variety of samples such as fluorescent dyes, liquid phantoms, and cervical tissue samples. The resultant polarized and intrinsic fluorescence spectra are in consonance with what is usually observed through commercially available spectrometers. In the future, our main motive is to use this device in clinics for in-vivo testing and perform an AI (artificial intelligence) based classification for different stages of cervical cancer. For easy handling and regular use as a screening device, we intend to develop an app with built-in camera facility to capture only the raw images and then design a cloud server-based interface to make it accessible in remote areas. In conclusion, it can be said that the proposed smartphone-based device has the potential to become a standalone tool for the early diagnosis of cervical cancer.

## Supplementary Information

Supplementary Information.

## Data Availability

Raw experimental data produced/analyzed during this study is currently not publicly available but can be provided by corresponding author upon reasonable request. All the Software/codes used in this manuscript are freely available and easily accessible.
