# Supplementary material for: Design, fabrication and testing of 3D printed smartphone-based device for collection of intrinsic fluorescence from human cervix
Source: Sci Rep. 2022 Jul 1;12:11192. doi: 10.1038/s41598-022-15007-x (PMC9249735; doi:10.1038/s41598-022-15007-x)
Supplement: Supplementary file 1 — Supplementary Information. [file 41598_2022_15007_MOESM1_ESM.pdf]

Design, fabrication and testing of 3D printed smartphone-based device for collection of intrinsic fluorescence from human cervix

Shivam Shukla, Amar Nath Sah, Diganta Hatiboruah, Shikha Ahirwar, Pabitra Nath, and Asima Pradhan

Procedure for wavelength calibration of the system

- Crop the captured images by selecting region of interest which should be equal for each image.
- Calculate Gray Scale Intensity (GSI) for each image using formula:

$$GSI = \frac{R + G + B}{3} \qquad -(S1)$$

where, R, G, B are red, green, and blue values of a particular pixel.

- Find out pixel with maximum GSI (using equation) for each laser source and also the corresponding central wavelength using spectrometer data.
- Now, use these pixel and wavelength values to calculate multiplication (MF) and constant factors (CF), using equations (S2) and (S3), for each visible wavelength window Violet-Blue, Blue-Green, Green-Red and convert intensity v/s pixel curve into intensity v/s wavelength curve.

$$MF = \frac{(\lambda_1 - \lambda_2)nm}{(P_1 - P_2)pixel} \qquad -(S2)$$

$$CF = \lambda_1 - \left(MF\left(\frac{nm}{pixel}\right) * P1\right) \qquad -(S3)$$

where, (λ_1, λ_2) are the central wavelengths and ($P1$, $P2$) are the pixels corresponding to maximum intensity.

Since, the response of CMOS is non-linear to input spectral intensity, we have used three different MFs for the calibration of our system for the entire visible range:

Violet-Blue: 0.2582, Blue-Green: 0.2648, and Green-Red: 0.2745 nm/pixel.
